# Difficult Lung Isolation in a Heavy Smoker With a History of Left Lower Lobectomy: A Case Report

**DOI:** 10.7759/cureus.69388

**Published:** 2024-09-14

**Authors:** Daryl Jian An Tan, Cynthia Ming Li Chia, Sophia Chew

**Affiliations:** 1 Anesthesiology and Perioperative Medicine, Singapore General Hospital, Singapore, SGP; 2 Cardiothoracic Surgery, Singapore General Hospital, Singapore, SGP

**Keywords:** bronchial distortion, cardiothoracic anaesthesia, case report, one lung ventilation, thoracic surgery

## Abstract

Anatomical changes in the bronchial tree following a left lower lung lobectomy are not well documented in the current literature. Understanding these changes is crucial for selecting the appropriate lung isolation device and achieving optimal single-lung ventilation. We present the case of a 60-year-old female with a history of left lower lobectomy who was scheduled for elective video-assisted thoracoscopic surgery. This case highlights the unique challenges and management strategies involved, aiming to raise awareness within the medical community.

## Introduction

There is a gap in the current medical literature regarding the anatomical changes in the bronchial tree following a left lower lung lobectomy. This lack of detailed understanding can make achieving adequate single-lung isolation challenging during surgery. Additionally, patients with prior lung resections often have reduced lung reserves [1], which underscores the importance of selecting the right lung isolation device. This choice is crucial for optimizing surgical exposure while preserving the patient’s pulmonary function. We present a case of difficult lung isolation complicated by persistent hypoxemia in a patient with a history of left lower lung lobectomy.

## Case presentation

**Figure 1:**
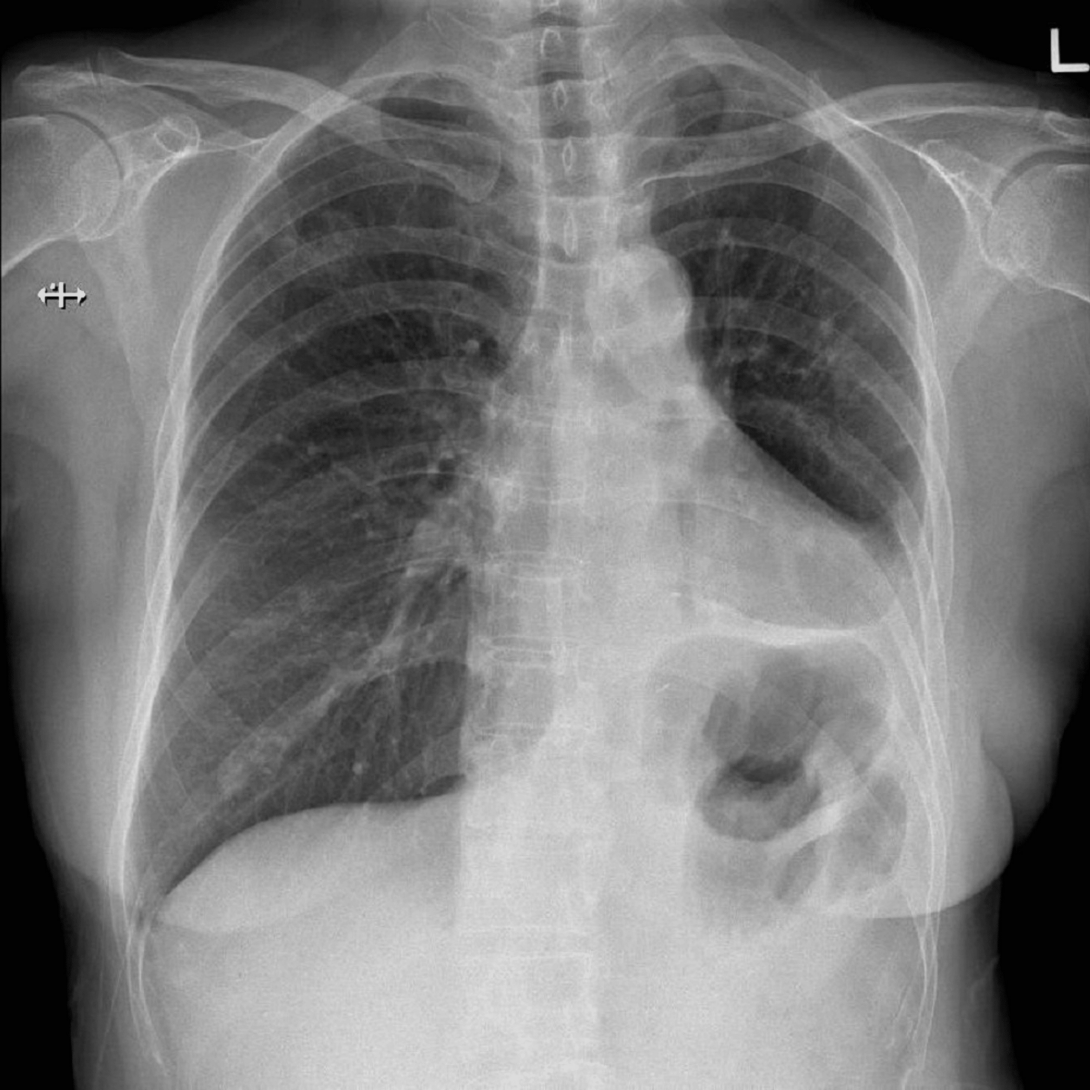
Preoperative chest X-ray image demonstrating mediastinal shift to the left, elevation of the left hemidiaphragm, and pleural thickening at the left costophrenic angle. An irregularly shaped opacity, measuring 2.6 cm, is visible in the right upper lung zone. This opacity was later confirmed by biopsy to be Stage 1 non-small cell lung cancer adenocarcinoma.

A 60-year-old Chinese female, with a height of 154 cm and weight of 51.3 kg, was scheduled for elective video-assisted thoracoscopic surgery to resect the right upper lung lobe. The procedure was planned due to a 2.8 cm sub-solid ground-glass lesion, which was biopsy-proven to be Stage 1 non-small cell lung cancer adenocarcinoma. She had a history of active smoking, totaling 40 pack-years, and had undergone a left lower lobectomy in 1996. Preoperative spirometry revealed FEV1 of 1.15 L (59%), FVC of 1.46 L (61%), FEV1/FVC ratio of 96%, and DLCO of 66%. Her preoperative chest X-ray is shown in Figure 1. At the time of preoperative assessment, the patient was in good health, with no signs of infection, and she had not smoked for 24 hours.

**Figure 2:**
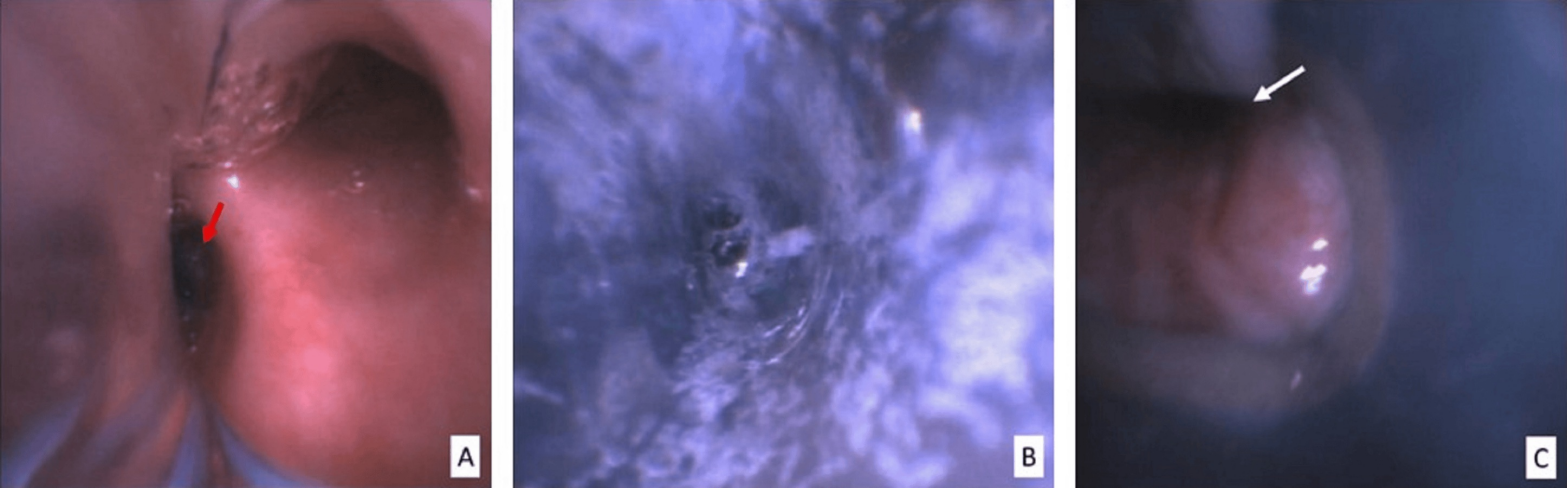
Bronchoscopy examination through the left-sided DLT after placement. (A) The view through the tracheal lumen shows proper placement of the DLT, with the carina visible and the bronchial lumen cuff inflated (red arrow) positioned in the left mainstem bronchus. (B) The view through the bronchial lumen reveals excessive secretions from the left lung. (C) The image displays the short and narrow left mainstem bronchus, with the tip of the bronchial lumen obstructing the opening of the LULB (white arrow). DLT, double-lumen tube; LULB, left upper lobe bronchus

After the induction of general anesthesia, a size 35 left-sided double-lumen tube (DLT) was inserted, and correct placement was confirmed by precordial auscultation and bronchoscopy. However, during the attempt to perform one-lung ventilation (OLV) of the left lung (non-operative), the peak airway pressures (Ppeak) exceeded 30 cmH_2_O, accompanied by a drop in end-tidal carbon dioxide (ETCO_2_) and hypoxemia, with SpO_2_ reaching a nadir of 82%. A subsequent bronchoscopy revealed excessive whitish secretions and a very short run-off to the left mainstem bronchus, causing the DLT bronchial lumen to partially occlude the inlet of the left upper lobe bronchus (LULB) (Figure 2). Despite performing bedside bronchoalveolar lavage and multiple attempts to reposition the DLT, OLV to the left lung remained unsustainable, preventing the safe continuation of the surgery.

A size 35 right-sided DLT was used instead, and OLV was initiated, significantly improving ventilation and oxygenation of the left lung. However, with only a single left lobe ventilated during OLV, maintaining patient saturation proved challenging. The lowest SpO_2_ readings dropped to 68% despite 100% inspired oxygen fraction (FiO_2_), requiring frequent lung recruitment and manual ventilation. Expiratory tidal volumes were poor, ranging from 160 to 180 ml, and Ppeak were between 35 and 38 cmH_2_O. Intermittent continuous positive airway pressure (CPAP) of 5 cmH_2_O was applied to the right (operative) lung to improve ventilation/perfusion (V/Q) matching, provided it did not interfere with the surgical view.

The patient tolerated a sub-lobar resection of the right upper lobe without lymphadenectomy. An arterial blood gas analysis at the end of the surgery, when the patient was placed back on two-lung ventilation with FiO_2_ of 0.6, showed a pH of 7.252, PaCO_2_ of 59.2 mmHg, and PaO_2_ of 168.0 mmHg. These results were consistent with high ETCO_2_ readings of 56-60 mmHg despite optimization of lung ventilation. Postoperatively, the patient was gradually weaned off the ventilator, transitioned to a single-lumen endotracheal tube, and successfully extubated after three hours.

## Discussion

Studies have shown that structural and functional alterations in the bronchial tree can occur after lobectomy [2]. These changes result from the adaptive remodeling of the remaining respiratory system following the procedure. If this remodeling decreases the angle between the trachea and the left mainstem bronchus, it can lead to distortion and narrowing of the remaining bronchus. This phenomenon is commonly described after left upper lobectomy [3] but is less reported following left lower lobectomy. Additionally, the reduced cross-sectional area increases airflow velocity and airway pressures in the tracheobronchial tree, contributing to elevated airway pressures observed in patients with a resected lobe. Notably, our case of a previous left lower lobectomy presented bronchoscopy findings typically associated with left upper lobectomy.

Managing hypoxemia during OLV is crucial in thoracic surgery. This patient had multiple risk factors for hypoxemia during OLV [4], including poor lung reserves, a significant smoking history, chronic lung disease, a prior pulmonary resection, and surgery on the right lung. Immediate management of emergent causes such as pneumothorax, bronchospasm, pulmonary hypertensive crisis, and cardiac failure is essential. Additionally, common issues like mal-positioning of the lung isolation device, anemia, anesthetic techniques inhibiting hypoxic pulmonary vasoconstriction, and inappropriate ventilatory settings for the dependent lung should be addressed. If hypoxemia persists, maneuvers like applying CPAP, low-flow oxygen insufflation, or intermittent two-lung ventilation [5] should be considered, although these can inflate the operative lung and compromise surgical exposure.

In our case, despite the proper placement of the left-sided DLT, the previous left lung resection caused a mediastinal shift and distorted anatomy, resulting in partial occlusion of the left LULB by the bronchial cuff of the DLT. Although issues with DLT placement and upper lobe ventilation are more common with a right-sided DLT due to the shorter right mainstem bronchus, our case presented similar challenges with the left-sided DLT due to the prior lower lobe resection, which is highly unusual. We decided to use a right-sided DLT since the right lung was to be collapsed and isolated for surgery, and only a sub-lobar resection of the upper lobe was performed. While a bronchial blocker could be used for lung isolation, it is prone to mal-positioning and does not allow for CPAP application to the operative lung, which was crucial for maintaining adequate oxygenation in this patient with poor lung reserves [6].

## Conclusions

Our case presented challenges due to the previous left lower lobectomy, which led to difficulties with DLT placement in a distorted left mainstem bronchus and complications with OLV in a patient with poor lung reserves. We present this case report to highlight the anatomical effects of a previous lower lobectomy on the tracheobronchial tree and to emphasize the implications of selecting the appropriate lung isolation device.
